# P02.69. Self administered acupuncture for treatment of chemotherapy associated nausea: a pilot study

**DOI:** 10.1186/1472-6882-12-S1-P125

**Published:** 2012-06-12

**Authors:** Z Adler, P Hansen

**Affiliations:** 1 University of Utah Department of Physical Medicine and Rehabilitation, Salt Lake City, USA

## Purpose

Acupuncture has gained popularity since the National Institutes of Health 1997 Consensus Statement concluded that acupuncture is an effective anti-emetic for adult postoperative and chemotherapy-induced nausea and vomiting. The most commonly used acupuncture point to control nausea and vomiting is the Pericardium 6 (P6), or Neiguan point. Some evidence suggests that the anti-emetic effects of P6 stimulation by acupuncture on chemotherapy-induced nausea and vomiting may last about eight hours. Most patients do not have access to a trained acupuncturist at such intervals. The purpose of this pilot study was to learn whether: 1) patients can be taught safe self-administration of acupuncture at P6 during cycles of their chemotherapy regimen and 2) self administered acupuncture reduces the severity of chemotherapy associated nausea and reduces use of anti-nausea medications.

## Methods

Twenty patients with chemotherapy associated nausea were recruited from Huntsman Cancer Hospital. Patients were randomized to groups A or B for a crossover trial. Patients were taught how to self-administer acupuncture at the P6 site. Acupuncture was self-administered a minimum of one and a maximum of three times per day during the first week of chemotherapy cycle #1 for group A and chemotherapy cycle #2 for group B. Acupuncture was used in conjunction with ongoing standard care. Both groups maintained daily logs documenting nausea on a scale of 1-10, emesis, medications used and time of acupuncture administration.

## Results

16 out of 20 patients successfully completed their daily logs. There was a small but statistically significant reduction in nausea severity during the acupuncture treatment cycle compared to control cycle. There was not a statistically significant reduction in episodes of emesis. There were no adverse events.

## Conclusion

Cancer patients can be safely taught self administration of acupuncture at P6 in order to reduce the severity of chemotherapy associated nausea.

